# Sexual dimorphism in the association between gestational diabetes mellitus and overweight in offspring at 5-7 years: The OBEGEST cohort study

**DOI:** 10.1371/journal.pone.0195531

**Published:** 2018-04-05

**Authors:** Nathalie Le Moullec, Adrian Fianu, Olivier Maillard, Emilie Chazelle, Nadège Naty, Chantal Schneebeli, Patrick Gérardin, Laetitia Huiart, Marie-Aline Charles, François Favier

**Affiliations:** 1 Department of Endocrinology, Diabetology and Nutrition, Centre Hospitalier Universitaire (CHU) Réunion, Saint-Pierre, Reunion, France; 2 INSERM CIC 1410 Clinical and Epidemiology/ CHU Réunion/Université de la Réunion, Saint-Pierre, Reunion, France; 3 UMR1027, Université de Toulouse, UPS, Inserm, Toulouse, France; 4 Center for Research in Epidemiology and Population Health, Paris, France; 5 Paris Descartes University, Paris, France; Mount Sinai Health System, University of Toronto, CANADA

## Abstract

Evidence from literature is mixed regarding a possible association of maternal gestational diabetes mellitus (GDM) and overweight in the offspring. Sexual dimorphism, or sex disparities in the pathogenesis linking GDM exposure to overweight, could be at play. The objective of this study was to investigate the association between GDM and child overweight at 5–7 years. Six hundred pairs (1:1) of Reunionese liveborn singletons selected from a hospital-based birth registry, matched for sex, gestational age, and birth period, underwent a prospective in-home follow-up and were analyzed with respect to their exposure to GDM. The primary outcome was child overweight at 5–7 years, as defined by the International Obesity Task Force. The association between GDM exposure and child overweight was estimated by the odds ratio (OR) using conditional logistic regression models. For the subset of children exposed to GDM with available maternal glycemic data, we analyzed the relationship between maternal glycemic levels during pregnancy and child body mass index (BMI) at 5–7 years with a linear regression model. In light of the significant interaction between sex and GDM, all statistical analyses were then stratified by sex. After controlling for pre-pregnancy BMI and maternal sociodemographic characteristics, the risk of overweight increased with exposure to GDM for boys (adjusted OR: 2.34; 95% confidence interval = 1.26–4.34, *P* = 0.007) but not for girls (adjusted OR: 0.56; 95%CI = 0.28–1.10, *P* = 0.093). Consistent with this, the linear increase of boys’ BMI at 5–7 years with maternal blood glucose levels during pregnancy, in the exposed group, displayed a dose-response relationship. Our findings indicate that exposure to GDM is a risk factor for childhood overweight in boys but not in girls, which adds to the growing body of evidence suggesting greater sensitivity of male offspring to intrauterine hyperglycemia.

## Introduction

Gestational Diabetes Mellitus (GDM) affects up to 16% of pregnancies [1], with considerable variations depending on the population characteristics and the diagnosis criteria used [2]. In recent years, changes in guidelines for GDM diagnosis, delayed motherhood and higher prevalence of obesity, along with increasingly unhealthy lifestyles have led to an upsurge of GDM [3,4].

GDM is associated with higher risks of adverse pregnancy outcomes for both the mother and the child. Maternal complications include preeclampsia, pregnancy-induced hypertensive disorders, and cesarean sections [5]. Maternal hyperglycemia increases levels of fetal glucose and causes hyperinsulinemia, leading to most fetal problems, collectively referred as diabetic fetopathy [6]. Indeed, newborn babies are at risk of macrosomia (large-for-gestational age) as a result of insulin and insulin-like growth factors that together stimulate fetal growth. This predisposes infants to shoulder dystocia and to adverse perinatal outcomes caused by abnormally-distributed fetal adiposity [7].

Furthermore, GDM significantly increases the risk of lifelong complications for both the mother and the child [8]. Studies have clearly shown that GDM is a strong risk factor for subsequent maternal type 2 diabetes (T2D) [9]. Intrauterine exposure to hyperglycemia has also been reported as a long-term risk factor for child obesity [10,11]. However, it is unclear whether hyperglycemia caused by GDM is independently associated with child overweight or not. Thus, recent systematic reviews and meta-analyses suggest inconsistent associations between GDM and the onset of overweight in childhood (> 1 year of age). So far these studies have been skewed not to include all relevant confounders, notably pre-pregnancy adiposity, which is a well-known risk factor for both GDM and child overweight [12,13]. Such discrepancies may also be explained by heterogeneity of age of the child at evaluation for obesity, and the hypothesis has been made that GDM exposure is temporarily linked to child adiposity [14,15]. Moreover, some studies suggest the presence of genomic imprinting in intrauterine programming and sexual dimorphism, i.e., the existence of sex disparities in child outcomes [16].

On Reunion Island, a French overseas department known to have both high prevalences of T2D and central adiposity [17], the OBEGEST cohort study was implemented to test the hypothesis of increased likelihood of adiposity among children exposed to GDM. The research objective was thus to assess the association between GDM and child overweight at 5–7 years. We did not take sexual dimorphism into account when designing our research; however, a post hoc analysis stratified by child sex has informed the study.

## Methods

### Setting, population and research design

**Fig 1:**
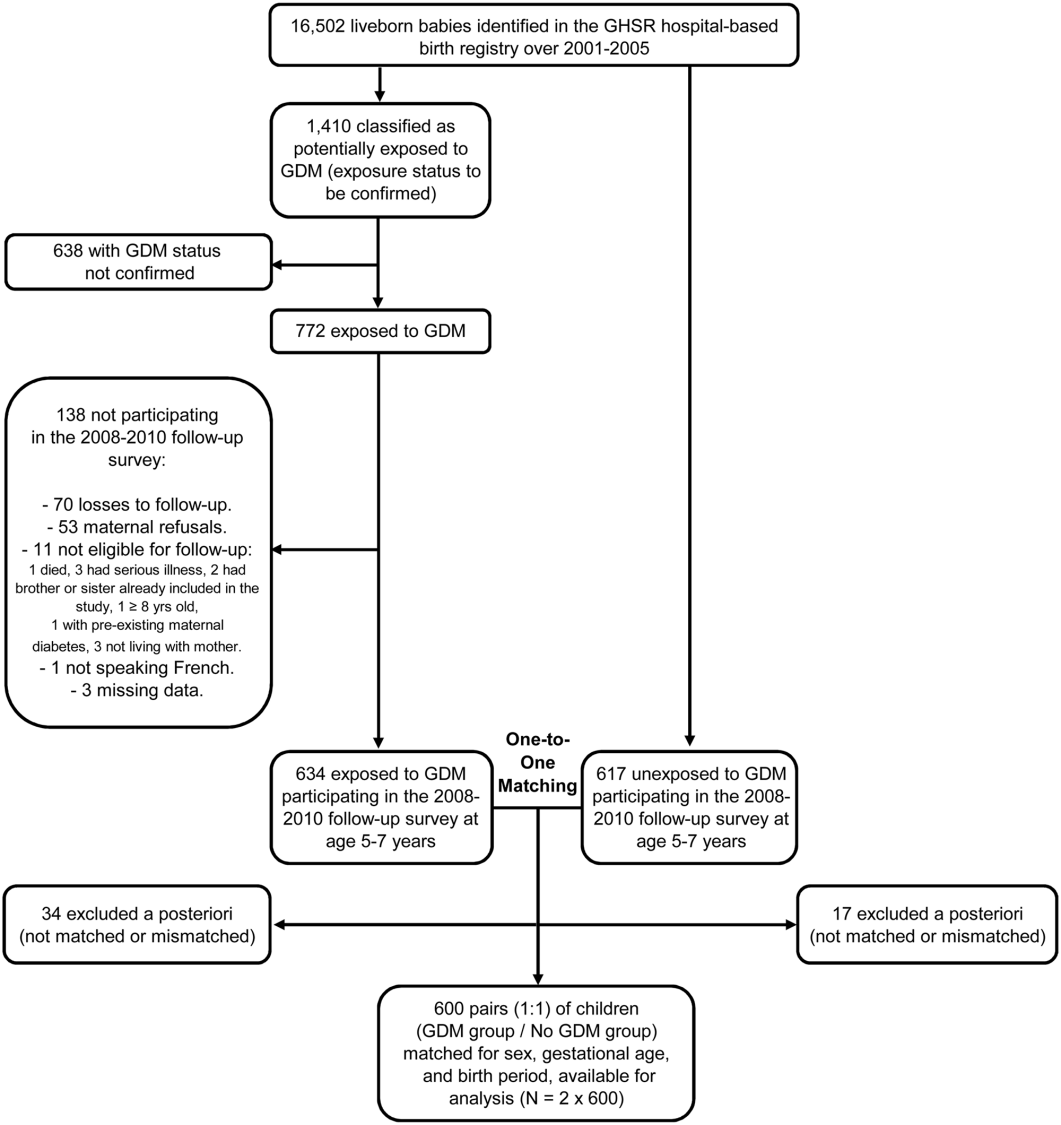
Selection of infants for the OBEGEST cohort study (South Reunion Island, 2001–2010). GDM: gestational diabetes mellitus. GHSR: Groupe Hospitalier Sud-Réunion.

This study was conducted on Reunion Island, a French overseas department of 800,000 inhabitants located in the Southwestern Indian Ocean. Reunion Island is known to have high prevalence rates of T2D (20.1% in adults 30–69 years), GDM (7.5%), overweight and obesity [17,18]. This health situation illustrates epidemiological transitions and westernization of lifestyles over the past few decades [17]. In the south of the island, the Groupe Hospitalier Sud-Réunion (GHSR) covered a population of 290,000 inhabitants, and its two participating maternity wards accounted for about 75% of births. Within these two maternity wards, over 2001–2005, all liveborn babies were retrospectively identified in the database of the GHSR birth registry, in order to be eligible for the OBEGEST cohort study (Fig 1).

First, we selected the singletons who had not been exposed to pre-existing maternal diabetes mellitus (type 1 or 2), and classified them as potentially exposed/unexposed to GDM using electronic medical records. Previous GDM was not an exclusion criterion. For a given mother, only the first child born during the 2001–2005 period was selected.

Then, for each child exposed to GDM (see definition below) and participating in the 2008–2010 follow-up survey (see description thereafter), we searched in the database for a *referent* [19] with the same sex, gestational age (as close as possible), and birth period (month/year), for appropriate One-to-One Matching (Fig 1). On average, up to three referent candidates could be available. For convenience and feasibility reason, we selected only and preferentially the first referent whose exposure status had been medically confirmed and mother had consented to participate in the follow-up survey.

Consequently, this selection process allowed to perform a *Matched Pairs Analysis* [19] of two groups of children (Fig 1): one exposed to GDM (named ‘GDM group’) versus another one not exposed to GDM (‘No GDM group’, also named ‘unexposed group’).

### Maternal antenatal characteristics

Maternal pre-pregnancy weight and height were the self-reported values collected during the first antenatal medical visit, available from the database of the GHSR birth registry. Pre-pregnancy body mass index (BMI) was categorized according to WHO cut-off values [20].

### Exposure status

GDM exposure status was confirmed after examination of alternative data sources (medical charts, hospital and laboratory electronic databases). Specifically, GDM status was established if the mother was screened positive based on a glucose challenge test (1-hr post load 50-g plasma glucose ≥ 11.1 mmol/l), had a diagnosis of GDM based on a 100-g oral glucose tolerance test (OGTT with at least two pathologic values defined as: fasting, ≥ 5.3 mmol/l; 1-hr, ≥ 10.0 mmol/l; 2-hr, ≥ 8.6 mmol/l; 3-hr, ≥ 7.8 mmol/l) [21], and/or had received insulin treatment during pregnancy. A small number of participants (< 0.5%; n = 6) with no available data were also classified into the GDM group if they combined high fasting (or postprandial) glycemic values with intense medical monitoring during pregnancy. Absence of GDM was defined by a negative screening test result or by a negative diagnosis of GDM based on OGTT (which excluded impaired glucose tolerance), regardless of screening result. A small number of patients (n = 4) who were given a screening test, but for whom no data was available in the medical charts, were included in the unexposed group, after a physician examination confirmed the absence of GDM risk factors (age ≥ 35 years old, overweight or obesity, first-degree family history of diabetes, personal history of GDM) and the presence of normal postprandial glycemic values (when available).

Ascertainment (i.e., the method by which persons with a trait are selected or discovered by an investigator) and follow-up survey participation rates were checked in both groups.

### Follow-up survey

Over the 2008–2010 period, we conducted a prospective in-home follow-up survey of the selected children at 5–7 years (Fig 1), in the chronologic order of their birth period. We made sure that the children were alive and were not severely-ill, and that they were still Reunion Island residents. Face-to-face questionnaires (251 items) were completed by trained dieticians during the in-home follow-up survey, with mothers providing information in the presence of their child. Data collection was about four main topics: socioeconomic and demographic characteristics, lifestyle of the child (e.g., diet, physical activity, sedentary lifestyle), anthropometric measures, and health conditions of mother and child.

The height of both child and mother was measured without shoes, using a stadiometer (Seca^™^) with an accuracy of 0.1 cm. Weight was measured with little clothing, using a calibrated electronic balance scale (Seca^™^) with an accuracy of 0.1 kg. BMI was calculated as weight divided by squared height (kg/meters). Waist circumference was measured using a tape to the nearest 0.1 cm, following WHO recommendations. In the absence of international cut-off values predetermined for age-specific waist circumference, an elevated waist circumference was defined according to age- and sex-specific medians, these latter being calculated in the unexposed group taken as referent group for any statistical comparison to the targeted population.

### Primary outcome

The primary outcome was child overweight (including obesity) at 5–7 years, as defined by the International Obesity Task Force (IOTF) [22]. Briefly, this definition lays on age- and sex-specific cut-off values for children BMI that are based on international data and linked to the WHO adult cut-off point of 25 kg/m^2^. For example, a 5.5-year old girl having a 17.20 kg/m^2^ BMI would pass through 25.00 kg/m^2^ BMI at age 18 and could thus be classified as overweight for her age.

### Study sample size calculation

The size of the cohort was established to detect a minimal odds ratio (OR) of 1.50 associated with GDM exposure and overweight at 5–7 years. This calculation was based on previous studies that reported OR values between 1.29 and 1.89 [23]. The other parameters used to calculate the sample size were an expected prevalence of child overweight at 6 years in the unexposed group set to 15% [24], a power of 80%, and a two-tailed alpha of 5%. Given these prerequisites, we aimed to include 690 children per group (GDM / No GDM). To account for matching, we updated this calculation using McNemar-Test-Power-Analysis in PASS 11 (NCSS, LLC. Kaysville, Utah, USA. www.ncss.com). Given an observed proportion of discordant pairs on primary outcome of 0.322, this update led to a sample size of 634 pairs. Calculation did not account for stratified analyses.

### Statistical analysis

Proportions were compared using Wald Chi-Square tests. Continuous data were compared using paired-samples Student t test or the Wilcoxon signed rank test, as appropriate. The association between GDM exposure and overweight at 5–7 years was estimated by the OR using conditional logistic regression models to account for matched data. Based on previous studies conducted in the field [25–27], some well-known confounding factors (maternal pre-pregnancy BMI, parity, maternal age and education) were adjusted for in multivariable models. First-order interaction terms were tested, and, accordingly, statistical analysis was stratified for variables associated with significant interactions at *P* ≤ 5%. To evaluate the consistency of the association between GDM exposure and child overweight at 5–7 years by sex, we performed a sensitivity analysis replacing the primary outcome by child obesity at 5–7 years as defined by the IOTF [22]. For the subset of children exposed to GDM with available glycemic data, we analyzed the relationship between maternal glycemic levels during pregnancy and child BMI at 5–7 years. This relationship was examined through a linear regression model using the 2h post-load glycemic value of 100-g-OGTT as the independent variable. In multivariable linear regression models, we included the child’s exact age (years) and squared exact age (years^2^) to account for a possible adiposity rebound in the shape of the relation between BMI and age of the child, after checking that beta coefficient for squared exact age was significantly different from 0 for both girls and boys. All the statistical analyses were carried out with SAS version 9.4 (SAS Institute Inc., Cary, NC, USA) excluding observation with missing data. Significance level for *P* values was set at 5%.

### Ethical considerations

This study followed the French law of bioethics and was approved by the regional ethics committee “CPP Sud-ouest et Outre Mer III” (2008-A00448-7). All mothers gave informed written consent and all children provide oral assent to participate in the research.

## Results

### Selection of participants

The selection process of the OBEGEST cohort is depicted in Fig 1. At the follow-up survey conducted 5 to 7 years after birth (mean delay: 6.3 years), we were able to trace 634 children in the GDM group (ascertainment rate = 54.8% [772/1,410]; participation rate = 82.1% [634/772]) and 617 children in the No GDM group (ascertainment and participation rates impossible to assess given matching). Out of these 1,251 children, 51 could not be matched to create an exposed/unexposed pair (34 children in the GDM group and 17 in the No GDM group). This selection process enabled us to create 600 pairs of children matched for sex, gestational age and birth period. For gestational age, we applied a stringent matching criterion as a large majority of children within pairs belonged to the same gestational week (94%), a minority (~5%) were similar given ± one week, and only a few (~1%) showed a difference of two weeks. In the GDM group, the perinatal characteristics of participants at follow-up survey were the same as those of non-participants—with the exception of geographical origin, since the number of mothers born on Reunion Island was higher among participants than among non-participants (92.4% *versus* 84.1%, *P* = 0.002).

At childbirth (Table 1), mothers in the GDM group were older (*P* <0.001) than those in the No GDM group; they also had higher parity (*P* <0.001) and were less educated (*P* <0.001).

**Table 1:** Characteristics of study subjects by offspring sex and gestational diabetes mellitus exposure group.

Socio-demographic, clinical and lifestyle characteristics	All		*P*	Boys		*P*	Girls		*P*
	GDM	No GDM		GDM	No GDM		GDM	No GDM	
	(n = 600)	(n = 600)		(n = 309)	(n = 309)		(n = 291)	(n = 291)	
**Mother**									
Primiparous	21.7	39.2	<0.001	21.7	42.2	<0.001	21.7	36.1	<0.001
Age (years) at delivery	32.4 ± 5.9	27.6 ± 6.2	<0.001	32.2 ± 6.1	27.2 ± 6.1	<0.001	32.6 ± 5.8	28.1 ± 6.3	<0.001
Education ^a^									
College	29.2	41.3	<0.001	31.4	42.9	0.003	26.9	39.6	<0.001
High school	28.7	32.2		26.1	32.8		31.5	31.6	
Elementary school	42.1	26.5		42.5	24.3		41.6	28.8	
Pre-pregnancy BMI (kg/m^2^)	27.0 ± 5.9	23.2 ± 5.0	<0.001	27.1 ± 6.1	23.1± 4.7	<0.001	27.0 ± 5.7	23.4 ± 5.3	<0.001
Pre-pregnancy BMI ≥ 25 kg/m^2^	58.0	27.0	<0.001	56.7	26.9	<0.001	59.3	27.2	<0.001
Gestational weight gain (kg)	8.6 ± 6.2	11.8 ± 5.6	<0.001	8.7 ± 5.6	11.8 ± 5.7	<0.001	8.6 ± 6.8	11.8 ± 5.6	<0.001
Weight gain (kg/m^2^) at follow-up since pregnancy (5–7 years after delivery)	1.7 ± 3.0	2.8 ± 3.1	<0.001	1.8 ± 2.9	2.9 ± 3.2	<0.001	1.6 ± 3.1	2.8 ± 3.0	<0.001
**Child**									
Birthweight (g)	3183 ± 563	3047 ± 500	<0.001	3217 ± 573	3108 ± 494	0.003	3146 ± 550	2981 ± 497	<0.001
Breastfeeding < 3 months	59.3	53.3	0.036	61.1	54.9	0.116	57.4	51.6	0.162
*Characteristics at follow-up (5–7 years after birth)*:									
Age (years)	6.12 (5.00–7.76)	6.29 (5.00–7.89)	<0.001	6.13 (5.00–7.70)	6.26 (5.00–7.84)	<0.001	6.11 (5.00–7.76)	6.31 (5.00–7.89)	<0.001
BMI ≥ IOTF-25	25.5	14.2	<0.001	29.2	12.3	<0.001	21.6	16.1	0.101
Waist circumference > median ^b^	53.2	44.3	0.002	54.9	44.4	0.007	51.4	44.1	0.104
Reported energy intake (kcal/day)	1615 ± 296	1604 ± 312	0.515	1682 ± 286	1679 ± 319	0.884	1543 ± 291	1525 ± 283	0.408
Time spent exercising during the week preceding follow-up									
≤ 0.75 hour	46.0	45.8	1.000	41.2	41.4	0.931	51.0	50.5	0.929
Time spent watching TV during the week preceding follow-up									
> 2 hours on school days	12.3	13.3	0.607	12.3	13.3	0.718	12.4	13.4	0.714

Figures are column percentage, mean ± standard deviation, median (min-max), and *P*value.

^a^ Education was tested for college / pre-college.

BMI: body mass index. GDM: gestational diabetes mellitus. IOTF: international obesity task force cut-off.

^b^ aged-sex specific cut-off in the unexposed group.

Moreover, they gained less weight during pregnancy (*P* <0.001), which was expected since the average pre-pregnancy BMI was higher in this group (*P* <0.001). In the GDM group, 41.2% of mothers received insulin treatment. In the overall cohort, median gestational age at childbirth was 38 weeks (min-max: 27–42 weeks) and sex ratio (boys/girls) was 1.06. Mothers in the No GDM group gained more weight than those in the GDM group following the index pregnancy (Table 1). The distribution of lifestyle factors investigated in the study (diet, physical activity, sedentary lifestyle) was similar in the two groups (Table 1). Twenty-five percent of children exposed to GDM were overweight (of whom 10.7% were obese) versus 14.2% (and 4.5%, respectively) in the No GDM group (Table 1).

Importantly, we found a significant interaction between GDM exposure and offspring sex for the risk of overweight at 5–7 years (p-value for the interaction term in adjusted model: *P* = 0.005). In light of this interaction, we stratified all further analyses by sex.

### Relationship between GDM exposure and child overweight at 5–7 years by sex

The most significant finding of our study was that the risk of overweight increased with exposure to GDM (versus No GDM) for boys but not for girls (Table 2).

**Table 2:** Gestational diabetes mellitus exposure and co-factors associated with offspring overweight (BMI ≥ IOTF-25) at 5–7 years by sex.

Conditional logistic regression	Exposure	Boys (309 pairs)			Girls (291 pairs)		
		OR	95% CI	*P*	OR	95% CI	*P*
**Crude models**							
GDM	No	1.00	-	<0.001	1.00	-	0.101
	Yes	3.13	1.97–4.95		1.41	0.94–2.13	
**Adjusted models**							
GDM	No	1.00	-	0.007	1.00	-	0.093
	Yes	2.34	1.26–4.34		0.56	0.28–1.10	
Maternal pre-pregnancy BMI	< 25 kg/m^2^	1.00	-	0.005	1.00	-	<0.001
	≥ 25 kg/m^2^	2.95	1.39–6.24		4.97	2.13–11.57	
Maternal status	Primiparous	1.00	-	0.481	1.00	-	0.571
	Multiparous	0.72	0.28–1.82		1.31	0.51–3.35	
Maternal age (continuous)	+ 5 years	1.13	0.78–1.64	0.510	1.09	0.79–1.52	0.598
Maternal education	College	1.00	-	0.752	1.00	-	0.233
	High school	1.18	0.47–2.98		0.84	0.34–2.09	
	Elementary school	0.81	0.33–2.01		1.78	0.64–4.95	

For each sex, two models are presented: one crude (non-adjusted) model and one adjusted model. Figures are odds ratios (OR), 95% confidence interval of odds ratios, *P*value for global effect. Data are from pairs (1:1) of children (exposed to GDM / unexposed to GDM) matched for sex, gestational age, and birth period. Reference category for dependent variable is BMI < IOTF-25. Missing data distribution (number of missing observations / total number of observations): Crude model for Boys (2/618); Crude model for Girls (0/582); Adjusted model for Boys (11/618); Adjusted model for Girls (13/582). BMI: body mass index. GDM: gestational diabetes mellitus. IOTF: international obesity task force cut-off.

When accounting for maternal characteristics (pre-pregnancy BMI, parity, age, education), GDM was associated with a significantly increased odds of child overweight among boys but not girls: adjusted OR for boys was 2.34 (95% CI = 1.26–4.34, *P* = 0.007) and adjusted OR for girls was 0.56 (95% CI = 0.28–1.10, *P* = 0.093).

In Table 2, maternal pre-pregnancy overweight (including obesity) was associated with child overweight at 5–7 years in both sexes.

The relationship between GDM exposure and child obesity at 5–7 years by sex is presented in S1 Table.

### Relationship between maternal hyperglycemia during pregnancy and child BMI at 5–7 years by sex

In the subset of children exposed to GDM with maternal glycemic measurement taken during pregnancy (Table 3), boys’ BMI at 5–7 years increased with levels of maternal glycemia measured 2 hours after 100-g-OGTT, after adjustment (*P* = 0.040). This relationship was non-significant for girls (*P* = 0.524).

**Table 3:** Maternal glycemic levels during pregnancy (mmol/l) related to offspring BMI (kg/m^2^) at 5–7 years by sex in group exposed to gestational diabetes mellitus.

Linear regression	Boys exposed to GDM				Girls exposed to GDM			
	n	Beta coefficient	(SE)	*P*	n	Beta coefficient	(SE)	*P*
Crude models	173	+0.27	(0.15)	0.070	168	+0.08	(0.15)	0.574
Adjusted models	169	+0.31	(0.15)	0.040	165	+0.09	(0.14)	0.524

For each sex, two models are presented: one crude (non-adjusted) model and one adjusted model. Data are from the subset (n) of children exposed to GDM with maternal glycemic measurement taken with oral glucose tolerance test (100-g-OGTT) available for analysis. The dependent variable is offspring BMI (kg/m^2^) at 5–7 years regressed by 2-h post-load maternal glycemic measurement (mmol/l). The co-factors included in the adjusted models were the same as in Table 2, plus the child’s exact age (years) and squared exact age (years^2^). BMI: body mass index. GDM: gestational diabetes mellitus.

## Discussion

After adjustment for confounding factors (in particular maternal pre-pregnancy BMI), the OBEGEST cohort study indicates that the risk of child overweight (including obesity) at 5–7 years increases with GDM exposure, but only in the male population. Consistent with this, the linear association between maternal blood glucose levels and boys’ BMI at 5–7 years in the GDM group may suggest a dose-response relationship: the more male offspring are exposed to maternal hyperglycemia, the higher their BMI. Importantly, our findings were observed whilst school-age visits revealed no significant difference in the lifestyle of children, which probably rules out a major influence of postnatal life events.

A few recent studies have noted the presence of sexual dimorphism in the relationship between child overweight and intrauterine exposure to GDM. Thus, in the Hong Kong center of the HAPO study (Hyperglycemia and Adverse Pregnancy Outcome) conducted in Chinese participants [28], female, but not male offspring of GDM mothers exhibited higher overall adiposity at 7 years than did offspring of normoglycemic mothers. However, the primary outcome of this study was abnormal glucose tolerance in the offspring, not the BMI. In a US cohort of children aged 7–8 years, an inverse relationship has been highlighted: as in the OBEGEST cohort study, only male offspring of GDM mothers presented increased adiposity [29]. The primary outcome of this latter was the Dual-Energy X-Ray Absorptiometry trunk-to-peripheral fat mass, which is a measure of central adiposity. Accordingly, a life course analysis of 15,000 US individuals followed-up through 2010 concluded that intrauterine exposure to GDM increases the risk of obesity among male only. This sex-specific association was found persistent from late childhood to early adulthood [30]. Consistent with these findings, our results show differential sensitivity of male and female fetuses to maternal hyperglycemia. This may be explained by the fact that male fetuses grow more quickly, need more nutrients (especially glucose), and are more insulin sensitive [31]. Conversely, maternal BMI was reported to be the primary predictor of adiposity in female but not in male neonates [32]. This latter finding is consistent with our adjusted estimates (Table 2), which suggest that the effect of maternal overweight on child overweight is stronger for girls (OR: 4.97) than it is for boys (OR: 2.95). However, it should be kept in mind that stratification on sex did not allow to test this comparison.

Recent studies have also shown that genes involved in appetite control and energy metabolism are epigenetically modified in the cord blood and placenta of infants from pregnancies complicated by GDM [33], which suggests a link between maternal hyperglycemia, fetal programming and long-term obesity. Furthermore, full-term placentas have been found to present sex-specific differences, including expression profiles for genes that regulate cell proliferation and hormone function [34].

GDM was recently shown to be associated with insulin, C-peptide, leptin, and growth factors (IGF-2 and IGFBP-3) in the cord blood of boys, but only with IGF-1 in the cord blood of girls [35]. A genome-wide DNA methylation analysis of donor pancreatic islets also revealed sex-specific differences in DNA methylation associated with changes in levels of relevant microRNAs, as well as with altered gene expression and insulin secretion [36]. This suggests differential programming in male and female fetuses, which reinforces the hypothesis that environmental intrauterine conditions may cause lifelong epigenetic changes.

Intrauterine exposure to maternal diabetes might result in earlier diagnosis of diabetes in offspring [37]. This is supported by siblings studies, which show that children born after their mother has developed a first episode of GDM exhibited higher glycated hemoglobin levels, BMI, and nearly fourfold odds of developing T2D than those born before an episode occurs [38]. Taken together, these findings highlight the challenges in distinguishing between the effects of genome-linked inherited risk factors and those associated with exposure to the intrauterine environment known as the epigenome—with both groups of factors constituting the fetal exposome [39].

### Study limitations

Our study has several limitations. First, analyses stratified by offspring sex may lack statistical power—especially in the case of girls—because they were not planned in the sample size calculation. Indeed, a posteriori statistical power calculations using the sex-specific estimates from the cohort led to an optimal level for boys (~100%) but only 34% for girls. The effect size is nonetheless clearly different between boys and girls, as evidenced by the significant interaction observed in our data. This interaction was supported by a sensitivity analysis performed using a more stringent definition of child adiposity (S1 Table). Second, the diagnostic criteria used by healthcare practitioners have changed since the OBEGEST study was completed, which has led to an expected two-fold increase in the prevalence of GDM [40]. This reinforces the relevance of our study for public health, even though questions persist. Third, insofar as longstanding interbreeding habits in Reunion Island have resulted in high genetic admixture for both males and females, we did not expect to find any difference based on ethnicity in the field; hence, we did not investigate this condition in our study. Fourth, we selected the 5 to 7-year age range because it precedes the physiological adiposity rebound in most children, which corresponds to the nadir of BMI growth. Thereby, we cannot exclude the possibility that the association between GDM and BMI at 5–7 years is short-lived and dependent on the child’s lifestyle. However, several studies suggest that long-lasting impact of environment and even epigenetic inheritance could be sex-specific [41]. Five, although the survey participation rate in exposed children was high, this could not be estimated in the unexposed group as the denominator was unknown given the selection process imposing One-to-One matching (Fig 1). For convenience, this selection process involved the first validated referent. Therefore, we cannot rule out a selection bias through the follow-up of the No GDM group, as observed within the GDM group. For consistency, we checked that the prevalence of child overweight in the unexposed group was equivalent to that estimated in a large regional study which was conducted at almost the same time, and which covered one third of Reunion Island children within the same age range: 14.3%, 95%CI = 13.5%-15.2%; N = 4,423 pupils aged 5.0–6.5 years [42]. Given the aforementioned, we believe that our matching strategy was adequate and selection is unlikely to have changed the overall sense of our findings. Another limitation of this study might be that we did not adjust for perinatal risk factors, including peripartum antibiotics and cesarean sections [43]. The latter are known to affect the composition of neonatal gut microbiota, which presumably influences child eating behavior [44]. Yet because they must be considered as intermediate factors in the causal chain between GDM and child overweight, they should not be controlled in the statistical analysis [45].

### Study strengths

First, the high prevalence of GDM in the population of Reunion Island facilitated enrolment in the cohort, while making this study especially relevant for public health. Second, the exhaustive database, which was completed prospectively, allowed us to identify children who were either exposed or unexposed to GDM. Third, access to multiple data sources helped to validate the exposure status of the children, blinded to primary outcome assessment. Fourth, in the GDM group, the 40% adherence rate to insulinotherapy may have reduced average fetus-exposure to maternal glycemia, resulting in a conservative association between GDM exposure and child overweight. Five, to minimize the selection bias compared with a hospital-based follow-up, we conducted a prospective in-home follow-up survey. The latter improved the quality of data especially self-reported data. Sixth, the assessment of children and mothers was performed by skilled dieticians in order to minimize evaluation bias. Lastly, the statistical models were adjusted for important confounding factors like maternal pre-pregnancy BMI making residual confounding very unlikely [12,13].

### Implications for research and public health

Finally, an increasing body of literature has examined the complex relationship between GDM and child overweight by studying phenotypes, genotypes and epigenotypes from birth to adulthood. Their conflicting findings can be partly explained by differences in outcomes, diet habits, adjustments and GDM definition used. Future research on the effects of GDM treatment is necessary to improve long-term outcomes in offspring and to confirm the significance of sexual dimorphism in child adiposity through different settings.

### Conclusions

Our findings indicate that exposure to GDM is a risk factor for childhood overweight, in boys but not in girls, and add to the growing body of evidence suggesting greater sensitivity of male offspring to intrauterine hyperglycemia.

## Supporting information

S1 File STROBE statement—Checklist of items that should be included in reports of cohort studies. (DOCX)

S2 File Tables of the pooled analyses combining girls’ and boys’ data. These two supplementary tables present results from the primary analyses of the OBEGEST cohort study conducted before the identification of the significant interaction between GDM exposure and offspring sex for the risk of overweight at 5–7 years.
(DOCX)

S3 File Survey questionnaire # 1 used in the study. In the original language (French).
(DOC)

S4 File Survey questionnaire # 2 used in the study. In the original language (French).
(XLS)

S5 File Survey questionnaire # 1 used in the study. English translation.
(DOC)

S6 File Survey questionnaire # 2 used in the study. English translation.
(XLS)

S1 Table Gestational diabetes mellitus exposure and co-factors associated with offspring obesity (BMI ≥ IOTF-30) at 5–7 years by sex. This table presents results from a sensitivity analysis performed using child obesity (instead of child overweight including obesity) as dependant variable in the conditional logistic regression models.
(DOCX)
