# Supplementary material for: Sexual dimorphism in the association between gestational diabetes mellitus and overweight in offspring at 5-7 years: The OBEGEST cohort study
Source: PLoS One. 2018 Apr 5;13(4):e0195531. doi: 10.1371/journal.pone.0195531 (PMC5886576; doi:10.1371/journal.pone.0195531)
Supplement: S1 File — (DOCX) [file pone.0195531.s001.docx]

STROBE Statement—Checklist of items that should be included in reports of ***cohort studies***

Sexual dimorphism in the association between gestational diabetes mellitus and overweight in offspring at 5-7 years:

the OBEGEST cohort study

|  | Item No | Recommendation | **Fulfilled** | **Location / Section / Subheading in text** |
| --- | --- | --- | --- | --- |
| **Title and abstract** | 1 | (*a*) Indicate the study’s design with a commonly used term in the title or the abstract | ⌧ | Title |
|  |  | (*b*) Provide in the abstract an informative and balanced summary of what was done and what was found | ⌧ | Abstract |
| Introduction | | |  |  |
| Background/rationale | 2 | Explain the scientific background and rationale for the investigation being reported | ⌧ | Introduction |
| Objectives | 3 | State specific objectives, including any prespecified hypotheses | ⌧ | At the end of the Introduction |
| Methods | | |  |  |
| Study design | 4 | Present key elements of study design early in the paper | ⌧ | Setting, population and research design |
| Setting | 5 | Describe the setting, locations, and relevant dates, including periods of recruitment, exposure, follow-up, and data collection | ⌧ | Setting, population and research design.  Follow-up survey.  Fig 1. |
| Participants | 6 | (*a*) Give the eligibility criteria, and the sources and methods of selection of participants. Describe methods of follow-up | ⌧ | Setting, population and research design.  Follow-up survey. |
|  |  | (*b*) For matched studies, give matching criteria and number of exposed and unexposed | ⌧ | Setting, population and research design.  Fig 1. |
| Variables | 7 | Clearly define all outcomes, exposures, predictors, potential confounders, and effect modifiers. Give diagnostic criteria, if applicable | ⌧ | Maternal antenatal characteristics.  Exposure status.  Follow-up survey.  Primary outcome. |
| Data sources/ measurement | 8* | For each variable of interest, give sources of data and details of methods of assessment (measurement). Describe comparability of assessment methods if there is more than one group | ⌧ | Maternal antenatal characteristics.  Exposure status.  Follow-up survey.  Primary outcome.  S3 File, S4 File, S5 File and S6 File. |
| Bias | 9 | Describe any efforts to address potential sources of bias  (matching, in-home follow-up, anthropometric measurement accuracy, age- and sex-specific cut-off values (IOTF) for BMI, adjustment in multivariate models) | ⌧ | Setting, population and research design.  Maternal antenatal characteristics.  Exposure status.  Follow-up survey.  Primary outcome.  Statistical analysis. |

|  | Item No | Recommendation | **Fulfilled** | **Location / Section / Subheading in text** |
| --- | --- | --- | --- | --- |
| Study size | 10 | Explain how the study size was arrived at | ⌧ | Study sample size calculation. |
| Quantitative variables | 11 | Explain how quantitative variables were handled in the analyses. If applicable, describe which groupings were chosen and why | ⌧ | Maternal antenatal characteristics.  Follow-up survey.  Primary outcome.  Statistical analysis. |
| Statistical methods | 12 | (*a*) Describe all statistical methods, including those used to control for confounding | ⌧ | Statistical analysis. |
|  |  | (*b*) Describe any methods used to examine subgroups and interactions | ⌧ | Statistical analysis. |
|  |  | (*c*) Explain how missing data were addressed | ⌧ | Statistical analysis. |
|  |  | (*d*) If applicable, explain how loss to follow-up was addressed | ⌧ | Fig 1. |
|  |  | (*e*) Describe any sensitivity analyses | ⌧ | Statistical analysis. |
| Results | | |  |  |
| Participants | 13* | (a) Report numbers of individuals at each stage of study—eg numbers potentially eligible, examined for eligibility, confirmed eligible, included in the study, completing follow-up, and analysed | ⌧ | Fig 1.  Selection of participants. |
|  |  | (b) Give reasons for non-participation at each stage | ⌧ | Selection of participants.  Fig 1. |
|  |  | (c) Consider use of a flow diagram | ⌧ | Fig 1. |
| Descriptive data | 14* | (a) Give characteristics of study participants (eg demographic, clinical, social) and information on exposures and potential confounders | ⌧ | Table 1.  Selection of participants. |
|  |  | (b) Indicate number of participants with missing data for each variable of interest | ⌧ | Legend of :  Table 2, S1 Table, first table of S2 File. |
|  |  | (c) Summarise follow-up time (eg, average and total amount) | ⌧ | Selection of participants. |
| Outcome data | 15* | Report numbers of outcome events or summary measures over time | ⌧ | Table 1 (%).  Selection of participants. |
| Main results | 16 | (*a*) Give unadjusted estimates and, if applicable, confounder-adjusted estimates and their precision (eg, 95% confidence interval).  Make clear which confounders were adjusted for and why they were included | ⌧ | Table 2.  Relationship between GDM exposure and child overweight at 5-7 years by sex. |
|  |  | (*b*) Report category boundaries when continuous variables were categorized | ⌧ | Table 2. |
|  |  | (*c*) If relevant, consider translating estimates of relative risk into absolute risk for a meaningful time period | Not Applicable | - |

|  | Item No | Recommendation | **Fulfilled** | **Location / Section / Subheading in text** |
| --- | --- | --- | --- | --- |
| Other analyses | 17 | Report other analyses done—eg analyses of subgroups and interactions, and sensitivity analyses | ⌧ | Relationship between maternal hyperglycemia during pregnancy and child BMI at 5-7 years by sex.  Table 3.  S2 File.  S1 Table. |
| Discussion | | |  |  |
| Key results | 18 | Summarise key results with reference to study objectives | ⌧ | First paragraph of the Discussion. |
| Limitations | 19 | Discuss limitations of the study, taking into account sources of potential bias or imprecision. Discuss both direction and magnitude of any potential bias | ⌧ | Study limitations. |
| Interpretation | 20 | Give a cautious overall interpretation of results considering objectives, limitations, multiplicity of analyses, results from similar studies, and other relevant evidence | ⌧ | Discussion. |
| Generalisability | 21 | Discuss the generalisability (external validity) of the study results | ⌧ | Implications for research and public health. |
| Other information | | |  |  |
| Funding | 22 | Give the source of funding and the role of the funders for the present study and, if applicable, for the original study on which the present article is based | ⌧ | Online registration. |

*Give information separately for exposed and unexposed groups.

**Note:** An Explanation and Elaboration article discusses each checklist item and gives methodological background and published examples of transparent reporting. The STROBE checklist is best used in conjunction with this article (freely available on the Web sites of PLoS Medicine at http://www.plosmedicine.org/, Annals of Internal Medicine at http://www.annals.org/, and Epidemiology at http://www.epidem.com/). Information on the STROBE Initiative is available at http://www.strobe-statement.org.
