# Supplementary material for: Sexual dimorphism in the association between gestational diabetes mellitus and overweight in offspring at 5-7 years: The OBEGEST cohort study
Source: PLoS One. 2018 Apr 5;13(4):e0195531. doi: 10.1371/journal.pone.0195531 (PMC5886576; doi:10.1371/journal.pone.0195531)
Supplement: S2 File — These two supplementary tables present results from the primary analyses of the OBEGEST cohort study conducted before the identification of the significant interaction between GDM exposure and offspring sex for the risk of overweight at 5–7 years. (DOCX) [file pone.0195531.s002.docx]

**- Tables of the pooled analyses combining girls’ and boys’ data -**

**Gestational diabetes mellitus exposure and co-factors associated with offspring overweight (BMI ≥ IOTF-25) at 5-7 years**

| **Conditional logistic regression** | **Exposure** |  | **OR** | **95% CI** | ***P*** |
| --- | --- | --- | --- | --- | --- |
|  |  |  |  |  |  |
| **Crude model** |  |  |  |  |  |
| GDM | No |  | 1.00 | - | <0.001 |
|  | Yes |  | 2.06 | 1.53-2.79 |  |
| **Adjusted model** |  |  |  |  |  |
| GDM | No |  | 1.00 | - | 0.384 |
|  | Yes |  | 1.20 | 0.80-1.81 |  |
| Maternal pre-pregnancy BMI | < 25 kg/m² |  | 1.00 | - | <0.001 |
|  | ≥ 25 kg/m² |  | 3.44 | 2.04-5.79 |  |
| Maternal status | Primiparous |  | 1.00 | - | 0.914 |
|  | Multiparous |  | 0.97 | 0.53-1.76 |  |
| Maternal age (continuous) | + 5 years |  | 1.12 | 0.89-1.40 | 0.344 |
| Maternal education | College |  | 1.00 | - | 0.778 |
|  | High school |  | 0.89 | 0.48-1.63 |  |
|  | Elementary school |  | 1.10 | 0.58-2.10 |  |

Figures are odds ratios (OR), 95 % confidence interval of odds ratios, *P*value for global effect. Data are from 600 pairs (1:1) of children (exposed to GDM / unexposed to GDM) matched for sex, gestational age, and birth period. Reference category for dependent variable is BMI < IOTF-25. Missing data distribution (number of missing observations / total number of observations): Crude model (2/1,200); Adjusted model (24/1,200). BMI: body mass index. GDM: gestational diabetes mellitus. IOTF: international obesity task force cut-off.

**Maternal glycemic levels during pregnancy (mmol/l) related to offspring BMI (kg/m²) at 5-7 years in the group exposed to gestational diabetes mellitus**

| **Linear regression** |  | **n** | **Beta coefficient** | **(SE)** | ***P*** |
| --- | --- | --- | --- | --- | --- |
| Crude model |  | 341 | +0.23 | (0.11) | 0.034 |
| Adjusted model |  | 334 | +0.25 | (0.11) | 0.015 |

Data are from the subset (n) of children exposed to gestational diabetes mellitus with maternal glycemic measurement taken with oral glucose tolerance test (100-g-OGTT) available for analysis. The dependent variable is offspring BMI (kg/m²) at 5-7 years regressed by 2-h post-load maternal glycemic measurement (mmol/l). The co-factors included in the adjusted model were the same as in Table 2, plus the child’s exact age (years) and squared exact age (years²). BMI: body mass index.
