# Supplementary material for: Sexual dimorphism in the association between gestational diabetes mellitus and overweight in offspring at 5-7 years: The OBEGEST cohort study
Source: PLoS One. 2018 Apr 5;13(4):e0195531. doi: 10.1371/journal.pone.0195531 (PMC5886576; doi:10.1371/journal.pone.0195531)
Supplement: S3 File — In the original language (French). (DOC) [file pone.0195531.s003.doc]

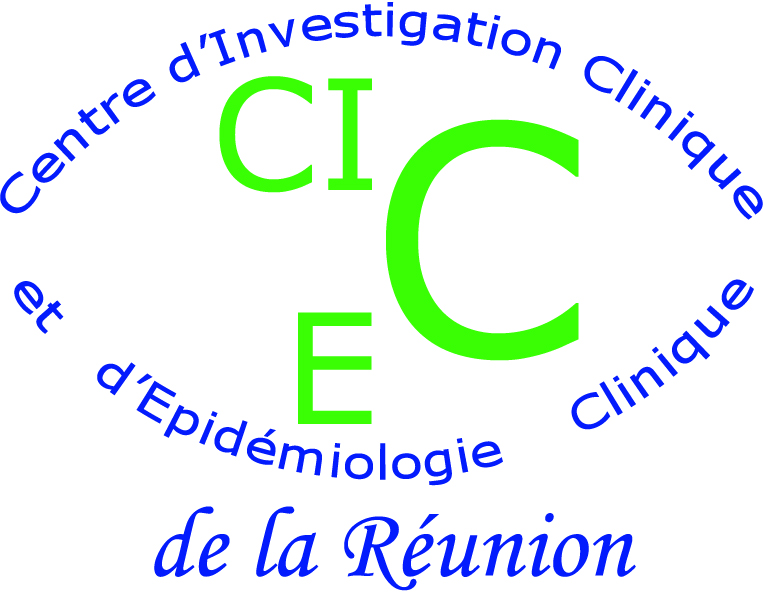


**OBEGEST**

**Enquête sur le diabète gestationnel et le risque d’obésité**

**chez l’enfant de 5-7 ans à la Réunion**

##### Identification de l’enfant et de la mère

## Enfant

Identifiant :(Num Id) **|__|__|__|__|**

Identifiant (Num): **|__|__|__|__|__|**

Nom : ……………………………………..................................................

Prénom : …………………………..............................................................

Sexe 1|__|M 2|__|F **|__|**

**Mère :**

Nom : ……………………………………..................................................

Prénom : …………………….………........................................................

Adresse : …………………………………………………………………

Téléphone fixe : …………………..............................................................

Téléphone mobile : …………………………........................................ ...

Date d’enquête : **|__|__| |__|__| |__|__|__|__|**

**Heure début d’enquête : |__|__|h**|__|__|**min**

Enquêtrice (initiales) 1**|__|__|**

Date d’enquête : 2**|__|__| |__|__| |__|__|__|__|**

**Identification de l’enfant**

Identifiant (NumId ou Num) : 3|__|__|__|__|__|

Sexe : 1|__|M 2|__|F 4|__|

Nombre de frères et sœurs biologiques : 5|__|__|

Rang de naissance : 6|__|__|

Date de naissance : 7|__|__| |__|__| |__|__|__|__|

Age au jour de l’enquête : 8|__|__| ans |__|__| mois

Lieu de naissance : 1|__|Saint-Pierre 2|__| Saint-Louis 9|__|

**Caractéristiques biométriques**

Le jour de l’enquête : Poids : 10|__|__|,|__| kg Taille : 11|__|__|__| cm

Tour de taille : 12|__|__|__| cm

A la naissance : Poids : 13|__|__|__|__| g

Taille  14**|__|__|__|** cm

Date : 15|__|__| / |__|__| /|__|__|__|__|

Vers l’âge de 9 mois : Poids : 16|__|__|kg |__|__|__| g Taille : 17|__|__|__| cm Date : 18|__|__| / |__|__| / |__|__|__|__|

Vers l’âge de 24 mois : Poids : 19|__|__|kg|__|__|__|g Taille : 20|__|__|__|cm Date : 21|__|__| / |__|__| / |__|__|__|__|

Vers l’âge de 4 ans : Poids : 22|__|__|, |__| kg Taille : 23|__|__|__| cm Date : 24|__|__| / |__|__| /|__|__|__|__|

**La Mère :**

Le jour de l’enquête :

Poids : 1|__|pesé 0|__|déclaré 25|__|

Résultats : 26|__|__|__| kg

Si enceinte, noter le poids déclaré ou mesuré avant la grossesse

Taille : 1|__|mesurée 0|__|déclaré 27|__|

Résultats : 28|__|__|__| cm

Tour de taille : 29|__|__|__|cm

**Le Père :**

Le jour de l’enquête :

Poids : 1|__|pesé 0|__|déclaré 30|__|

Résultats : 31|__|__|__|kg

Taille : 1|__|mesurée 0|__|déclarée 32|__|

Résultats : 33|__|__|__**|** cm

Tour de taille : 34**|__|__|__|** cm

**Consommations et préférences alimentaires de l’enfant**

#### Enquête de fréquence semi-quantitative sur les 15 derniers jours

Ration calorique quotidienne estimée 35|__|__|__|__| kcal/j

Répartition en macronutriments :

Glucides : 36**|__|__|** %

Lipides : 37**|__|__|** %

Protides : 38**|__|__|** %

**Les habitudes alimentaires de votre enfant**

Est-ce que votre enfant mange en dehors des repas et des goûters ?

0|__|Non 1|__| Oui 2|__| NSP 39|__|

Si oui, détailler en clair (boissons sucrées,...): ................................. 40|__|

................................................................................................................................

Que mettez vous comme goûter dans son cartable ? ............................................. 40a|__|

.................................................................................................................................

Est-ce que votre enfant mange à la cantine le midi ?

0|__| Non 1|__| Oui 2|__| NSP 40b|__|

Si oui, préciser le nombre de jour/semaine: 41|__|__|

Combien de fois par semaine votre enfant mange-t- il en dehors de la maison

à part la cantine ?

0|__| Aucune 1 |__| 1 à 2 fois 2|__| + 2 fois 3|__| NSP 42|__|

D’une façon générale, diriez-vous que votre enfant :

0|__| N’a pas souvent faim

1|__| A un appétit normal pour un enfant de son âge

2|__| A toujours faim, demande toujours à manger

3|__| NSP 43|__|

Quels sont ses goûts préférés ?

1|__| Sucrés 2|__| Salés

3|__| Les deux 4|__| NSP 44|__|

Quel est le temps passé à table en moyenne/jour (en minutes) ? ........................

................................................................................................................................... 45|__|__|__| mn

Est ce que les repas du week-end sont différents (plus riches, plus copieux) ?

0|__| Non 1|__| Oui 2|__| NSP 46|__|

Est ce qu’il se ressert systématiquement ?

0|__| Non 1|__| Oui 2|__| NSP 47|__|

Est ce qu’il suit un régime alimentaire particulier prescrit par un médecin ?

0|__| Non 1|__| Oui 2|__| NSP 48**|__|**

Si oui, précisez : ……………………………………………………… 49|__|

**Histoire alimentaire de l'enfant**

Type d'allaitement ?

1|__| Sein 2|__| Biberon uniquement

3|__| Mixte 4|__| NSP 50|__|

Si biberon exclusivement, la raison de ce choix (en clair) : ......................... 51|__|

...................................................................................................................................

La durée de l'allaitement maternel  (exclusif ou mixte): |__|__|,|__|Durée 52|__|__|

en 1|__| Semaine 2|__| Mois 3|__| An 53|__|

Avez-vous ajouté de la farine dans le biberon ?

0|__| Non 1|__| Oui 2|__| NSP 54|__|

A quel âge avez-vous introduit des légumes et des fruits ?

|__|NSP 55|__|__| mois

A quel âge avez-vous introduit des viandes, des poissons et des œufs ?

|__|NSP 56|__|__| mois

A quel âge, a-t-il commencé à prendre le même repas que la famille ?

|__| NSP 57|__|__| mois

# **Renseignements sur l'alimentation de la famille**

Nombre total de personnes qui mangent en moyenne à la maison : 58|__|__|

Nombre d’enfant de moins de 10 ans : 59|__|__|

Nombre de litres d'huile consommés par mois : 60|__|__|,|__|

Nombre de kg de sucre consommés par mois : 61|__|__|,|__|

Est ce que les repas sont pris devant la TV ?

1|__| Jamais 2|__| Rarement 3|__| Souvent 4|__| Très souvent 62|__|

Respectez-vous des principes alimentaires liés à la religion ?

0|__| Non 1|__| Oui 2|__| NSP 63|__|

Si oui, est-ce que votre enfant respecte les mêmes principes

alimentaires que vous? 0|__| Non 1|__| Oui 64|__|

Y a-t-il des allergies alimentaires dans la famille ?

0|__| Non 1|__| Oui 2 |__| NSP 65|__|

Si oui, lesquelles (aliments exclus par la famille) ? ……………… 66|__|

................................................................................................................................

................................................................................................................................

**activité physique de l’enfant**

**Soulignez dans le tableau ci-dessous toutes les activitées que l’enfant a effectués plus de 10 fois au cours de l’année précédente,  puis estimer le temps passé par l’enfant pour chacune des activités soulignée ?** (Ne pas inclure les activités scolaires)

| Activité | Nb mois /an | Nb fois / semaine | Nb heures / séance |
| --- | --- | --- | --- |
| Basket-ball |  |  |  |
| Bicyclette |  |  |  |
| Foot-ball |  |  |  |
| Moringue |  |  |  |
| Danse |  |  |  |
| Gymnastique |  |  |  |
| Hand-ball |  |  |  |
| Marche-randonnée |  |  |  |
| Equitation |  |  |  |
| Arts martiaux |  |  |  |
| Ping pong |  |  |  |
| Skate |  |  |  |
| Roller |  |  |  |
| Escalade |  |  |  |
| Rugby |  |  |  |
| Tennis |  |  |  |
| Natation club |  |  |  |
| Natation loisir |  |  |  |
| Volley ball |  |  |  |
| Surf |  |  |  |
| Autre activité |  |  |  |

Combien d'heures par semaine votre enfant a-t-il pratiqué des activités

sportives :

- au cours de la semaine précédente 67|__|__|,|__| h/sem

- au cours de l’année précédente 68|__|__|,|__| h/sem ***(ATTENTION : on exclut le temps passé à marcher et le temps passé en cours de sport à l’école)***

Au cours d’une journée normale, combien d’heures votre enfant passe t-il

habituellement à :

- dormir pendant la nuit  69|__|__|h/jour

- faire la sieste dans la journée 70|__|__|h/jour  ***-*** lire, dessiner, jeux calmes (jouer aux cubes, poupées...) 71|__|__|h/jour

Pendant une semaine typique, combien d’heures par jour votre enfant

passe-t-il à regarder la télévision ou à jouer à des jeux vidéos ?

**-** un jour de semaine 72|__|__|h/jour

- un jour de week-end 73**|**__|__|h/jour

###### Au cours des 12 derniers mois, votre enfant a-t-il joué dans des équipes de

sports collectifs (football, basket ball, …) ?

0|__| Non 1|__| Oui 2|__| NSP 74|__|

Si oui, nombre d’équipes : ………………………………………… 75|__|__|

Votre enfant a-t-il déjà été immobilisé au lit ou sur une chaise pendant

plus de 2 semaines suite à une maladie ou à un accident ?

0|__| Non 1|__| Oui 2|__| NSP 76|__|

Si oui, à quel âge ? 77|__|__|mois 78|__|__|,|__| an

Si oui, combien de temps a duré cette immobilisation ? 79|__|__|mois 80|__|__|an

Au cours de l’année écoulée, votre enfant a-t-il fréquenté un centre

de loisirs, le mercredi ou pendant les vacances ?

0|__| Jamais 1|__| Occasionnellement

2|__| Régulièrement 3|__| Systématiquement 81|__|

**Transport scolaire**

Comment l'enfant se rend t-il à l'école ?

1|__| En bus 2|__| En voiture 4|__| A pied

8|__| A vélo 16|__| Autre (plusieurs choix sont possibles) 82|__|__|

###### Si autre (préciser).......................................................................................... 83|__|

Si à vélo, ou à pied, combien de temps dure le trajet : 84|__|__|__| mn

Quelle distance est parcourue : 85|__|__| km

santé de l’enfant et de la mère

**La santé de l’enfant**

Votre enfant a-t-il une ou plusieurs maladie (s) chronique (s) (carie dentaire)?

0|__| Non 1|__| Oui 2|__| NSP 86|__|

Si oui, la (les) quelle (s) :............................................................................ 87|__|

.......................................................................................................................

Prise régulière de médicaments ?

0|__| Non 1|__| Oui 2|__| NSP 88**|__|**

Si oui, le (les) quel (s) : …………………………………………………. 89|__|

.................................................................................................................................

###### Y a-t-il des diabétiques dans la famille proche de votre enfant ?

0|__| Non 1|__| Oui 2|__| NSP 90**|__|**

Si oui :

1|__| Père biologique 2|__| Mère

4|__| Frère ou sœur 8 |__| Autres à précisez : .....................

....................................................................................................................... 91|__|

HbA1c réalisé : 0 |__| Non 1 |__| Oui 92**|__|**

Résultats : 93**|__|__|,|**__|%

**La santé de la mère**

Etes-vous diabétique ?

0|__| Non 1|__| DT1 2|__| DT2 3|__| NSP 94**|__|**

Si oui, quel est votre traitement actuel :

1|__| régime 2|__| comprimés 4|__| insuline 95|__|

Age d’apparition du diabète**:** 96**|__|__|** ans

Avez-vous des antécédents familiaux de diabète ?

0|__| Non 1|__| Oui 2|__| NSP 97|__|

Si oui : 1|__| Père 2|__| Mère

4|__| Frère ou sœur 8|__| Autre (précisez)  98|__|

.............................................................................................................................. 99|__|

ATTENTION : il peut y avoir plusieurs choix possibles

Est-ce que vous avez eu un diabète gestationnel?

0|__| Non 1|__| Oui 2|__| NSP 100|__|

Si oui, pour quelle(s) grossesse(s)

1|__| Grossesse de l'enfant enquêté seulement

2|__| Grossesse(s) antérieure(s) à celle de l'enfant enquêté

4|__| Grossesse(s) postérieure(s) à celle de l'enfant enquêté

8|__| Toutes les grossesses

16|__| NSP 101|__|__|

Si oui, pour combien de grossesses (G) |__|__| |__| NSP 102|__|__|

Si oui, traitement :

G1 : 0|__|non 1|__|régime seul 2|__|insuline 3|__| NSP 103|__|

G2 : 0|__|non 1|__|régime seul 2|__|insuline 3|__| NSP 104|__|

G3 : 0|__|non 1|__|régime seul 2|__|insuline 3|__| NSP 105|__|

G4 : 0|__|non 1|__|régime seul 2|__|insuline 3|__| NSP 106|__|

G5 : 0|__|non 1|__|régime seul 2|__|insuline 3|__| NSP 107|__|

G6 : 0|__|non 1|__|régime seul 2|__|insuline 3|__| NSP 108|__|

G7 : 0|__|non 1|__|régime seul 2|__|insuline 3|__| NSP 109|__|

G8 : 0|__|non 1|__|régime seul 2|__|insuline 3|__| NSP 110|__|

Avez-vous d’autre (s) maladie (s) chronique (s)?

0|__| Non 1|__| Oui 2|__| NSP 111|__|

Si oui, la (les) quelle (s) : ……………………………………………… 112|__|

Prise régulière de médicaments ? :

0|__| Non 1|__| Oui 2|__| NSP 113|__|

Si oui lesquels (autres qu’anti-diabétique, si diabète) : ……………………………………………………………………… 114|__|

HbA1c réalisée : 0|__| Non 1|__| Oui 115|__|

Résultats : 116**|__|__|,|**__|%

**Caractéristiques sociodémographiques**

**Vie et mode de garde de l’enfant**

Votre enfant vit-il avec ses deux parents :

0|__| Non 1|__| Oui 117|__|

Si non :

1|__| Mode de garde alternée 2|__| Chez sa mère

3|__| Chez son père 4|__| Chez une autre personne 118|__|

Votre enfant vit-il avec ses grands-parents ? 0|__| Non 1|__| Oui 119|__|

Si oui, les repas sont ils préparés par les grands parents ?

0|__| Non 1|__| Oui 2|__| NSP 120|__|

Votre enfant est-il scolarisé ?

0|__| Non 1|__| Oui 2|__| NSP 121|__|

En quelle classe est-il au moment de l’enquête ?

0|__| GS 2|__| CP 3|__| CE1 4|__|Autre 122|__|

Si autre à préciser : :............................................................................. 123|__|

**Caractéristiques de la mère**

Date de naissance de la mère : 124|__|__|/|__|__|/|__|__|__|__|

Nbre d’enfants de la mère  125**|__|__|**

Lieu de naissance de la mère :

1|__| Réunion 2|__| Métropole 3|__| AutreOI

4|__|Autre 5|__| NSP 126|__|

Dernière classe fréquentée : …………………….............................................. 127|__|

Age de fin d’études **:** 128**|__|__|**ans

Est-ce que vous avez un emploi : 0|__| Non 1|__| Oui 129|__|

Si oui, lequel : ........................................................................................ 130|__|

Combien d’heures/sem. : 131**|__|__|**h/sem

Si non, quel est votre statut : ................................................................. 132|__|

Si non, dernière profession exercée : .............................................. 133|__|

Quelle est ou était l'activité professionnelle de votre père :

............................................................................................................................ 134|__|

Quelle est ou était l'activité professionnelle de votre mère :

............................................................................................................................ 135|__|

**Caractéristiques du père de l’enfant**

Date de naissance du père : 136|__|__|/|__|__|/|__|__|__|__|

Lieu naissance du père :

1|__| Réunion 2|__| Métropole 3|__| Autre OI

4|__| Autre 5|__| NSP 137|__|

Dernière classe fréquentée : ............................................................................ 138|__|

Age de fin d’études : 139**|__|__|**

Est-ce que vous avez un emploi : 0|__| Non 1|__| Oui 140|__|

Si oui, lequel : ........................................................................................ 141|__|

Si non, quel est votre statut : ................................................................ 142|__|

Si non, dernière profession exercée : .................................................... 143|__|

Quelle est ou était l'activité professionnelle de votre père :

............................................................................................................................ 144|__|

Quelle est ou était l'activité professionnelle de votre mère :

............................................................................................................................ 145|__|

**Caractéristiques de la famille**

Actuellement, y a-t-il un fumeur au domicile ? 0|__| Non 1|__| Oui 146|__|

Quel est le type d’habitat ? 1|__| Individuel 2|__| Collectif 147|__|

A combien estimez vous votre budget mensuel réservé à l’alimentation : 148|__|__|__|__| euros

Quel est le revenu de la famille/mois : 149|__|

1|__| moins de 400 euros,

2|__| de 400 à moins de 800 euros,

3|__| de 800 à moins de 1200 euros,

4|__| de 1 200 à moins de 2000 euros,

5|__| de 2 000 à moins de 4 000 euros,

6|__| de 4 000 à moins de 6 000 euros,

7|__| 6 000 euros ou plus,

8|__| je ne sais pas,

9|__| je préfère ne pas répondre

**Heure fin d’enquête : |__|__|h|__|__|min**

Merci beaucoup d’avoir accepté de répondre à ce questionnaire.

Avez-vous des remarques à formuler : ------------------------------------------------------------------------------------------------------------------------------------------------------------------------------------------------------------------------------------------------------------------------------------------------------------------------------------------------------------------------------------------------------------------------------------------------------------------------------------------------------------

Observation personnelle de l’enquêtrice (difficulté à réaliser l’entretien...) :

-------------------------------------------------------------------------------------------------------------------------------------------------------------------------------------------------------------------------------------------------------------------------------------------------------------------------------------------------------------------------------------------------------------------------------------------------------------------------------------------------------------------------------------------------
