# Supplementary material for: Sexual dimorphism in the association between gestational diabetes mellitus and overweight in offspring at 5-7 years: The OBEGEST cohort study
Source: PLoS One. 2018 Apr 5;13(4):e0195531. doi: 10.1371/journal.pone.0195531 (PMC5886576; doi:10.1371/journal.pone.0195531)
Supplement: S5 File — English translation. (DOC) [file pone.0195531.s005.doc]

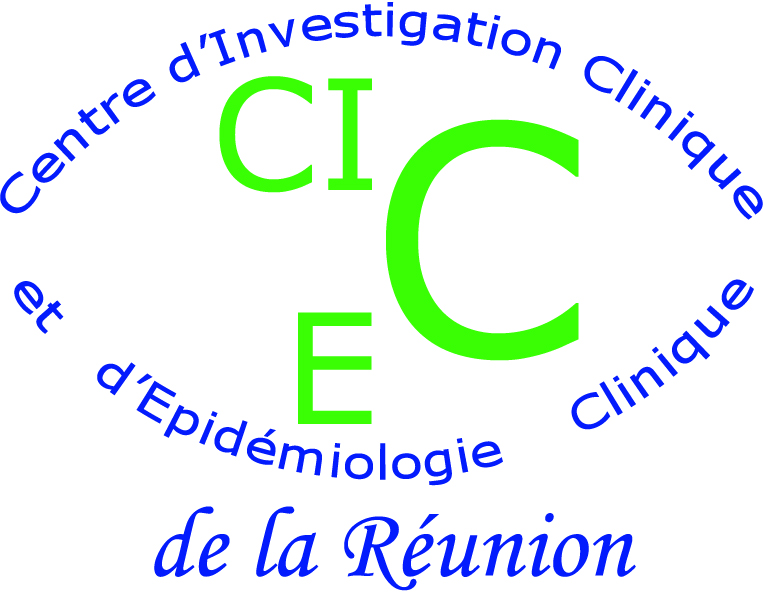


**OBEGEST**

**Survey of gestational diabetes mellitus and obesity risk in the child of 5-7 years on Reunion island**

##### IDENTIFICATION OF THE CHILD AND THE MOTHER

## Child

Identification (Num Id) **|__|__|__|__|**

Identification  (Num): **|__|__|__|__|__|**

Name: ……………………………………......................................................

First name: …………………………..............................................................

Sex 1|__|M 2|__|F **|__|**

**Mother:**

Name: ….……………………………………..................................................

First name: …………………….………..........................................................

Address: ……………………………………….………………………………

Phone number: ………………..……...............................................................

Cellular: …………………………………………...........................................

Date of survey: **|__|__| |__|__| |__|__|__|__|**

**Time at the start of the survey: |__|__|hour** |__|__|**minutes**

Investigator (initials) 1**|__|__|**

Date of survey: 2**|__|__| |__|__| |__|__|__|__|**

**IDENTIFICATION OF THE CHILD**

Identification  (NumId or Num)  3|__|__|__|__|__|

Sex: 1|__|M 2|__|F 4|__|

Number of biological brothers and sisters: 5|__|__|

Birth rank: 6|__|__|

Birth date: 7|__|__| |__|__| |__|__|__|__|

Age on the day of the survey: 8|__|__| year |__|__| month

Birth place: 1|__|Saint-Pierre 2|__| Saint-Louis 9|__|

**biométric Charactéristics**

The day of the survey: Weight: 10|__|__|.|__| kg Height: 11|__|__|__| cm

Waist circumference: 12|__|__|__| cm

At birth: Weight: 13|__|__|__|__| g

Height: 14**|__|__|__|** cm

Date: 15|__|__| / |__|__| /|__|__|__|__|

Around 9 months: Weight: 16|__|__|kg |__|__|__| g Height: 17|__|__|__| cm Date: 18|__|__| / |__|__| / |__|__|__|__|

Around 24 months: Weight: 19|__|__|kg|__|__|__|g Height: 20|__|__|__|cm Date: 21|__|__| / |__|__| / |__|__|__|__|

Around 4 years: Weight: 22|__|__|.|__| kg Height: 23|__|__|__| cm Date: 24|__|__| / |__|__| /|__|__|__|__|

**Mother:**

The day of the survey:

Weight: 1|__|measured 0|__|reported 25|__|

Results: 26|__|__|__| kg

If pregnant, write the reported or measured weight the pregnancy

Height: 1|__|measured 0|__|reported 27|__|

Results: 28|__|__|__| cm

Waist circumference: 29|__|__|__|cm

**Father:**

The day of the survey:

Weight: 1|__|measured 0|__|reported 30|__|

Results: 31|__|__|__|kg

Height: 1|__| measured 0|__|reported 32|__|

Results: 33|__|__|__**|** cm

Waist circumference: 34**|__|__|__|** cm

**diet and food preferences of the child**

#### Semi-quantitative frequency survey over the last 15 days

Estimated daily energy intake 35|__|__|__|__| kcal/j

Distribution of macronutriments:

Carbohydrates: 36**|__|__|** %

Lipids: 37**|__|__|** %

Proteins: 38**|__|__|** %

**The eating habits of your child**

Does your child eat outside meals’ hours?

0|__|No 1|__|Yes 2|__|Do not know 39|__|

If yes, specify (soft drinks, …): ................................................................. 40|__|

.................................................................................................................................

What snack do you put in his schoolbag? ………….............................................. 40a|__|

.................................................................................................................................

Does your child eat in the canteen at lunchtime?

0|__| No 1|__| Yes 2|__| Do not know 40b|__|

If yes, specify the number of days per week: 41|__|__|

How many times a week does your child eat outside the house except for the canteen?

0|__| Never 1 |__| 1 to 2 times 2|__| 2 times and more 3|__| Do not know 42|__|

In general, would you say that your child:

0|__| Is not often hungry

1|__| Has a normal appetite for a child of his age

2|__| Is always hungry, always asking to eat

3|__| Do not know 43|__|

What are his favorite tastes?

1|__| Sweet tastes 2|__| Salted tastes

3|__| Both 4|__| Do not know 44|__|

What is the average table time per day (in minutes)? ……………….......................

................................................................................................................................... 45|__|__|__| mn

Are the weekend meals different (richer, more hearty)?

0|__| No 1|__| Yes 2|__| Do not know 46|__|

Does your child take a second plate/portion systematically?

0|__| No 1|__| Yes 2|__| Do not know 47|__|

Does he follow a particular medical diet?

0|__| No 1|__| Yes 2|__| Do not know 48**|__|**

If yes, specify: ………………………………………….………………… 49|__|

**Diet history of the child**

Type of nursefeeding?

1|__| Breastfeeding 2|__| Only baby bottle

3|__| Mixed 4|__| Do not know 50|__|

If only baby bottle used, give the reason of this choice: .............................. 51|__|

...................................................................................................................................

Maternal nursefeeding duration (breastfeeding or mixed): |__|__|.|__|Duration 52|__|__|

in 1|__| Weeks 2|__| Months 3|__| Years 53|__|

Did you add flour to the baby bottle?

0|__| No 1|__| Yes 2|__| Do not know 54|__|

At what age did you introduce vegetables and fruits?

|__| Do not know 55|__|__| month

At what age did you introduce meat, fish and eggs?

|__| Do not know 56|__|__| month

At what age did your child take the same meal than the family?

|__| Do not know 57|__|__| month

# **Family feeding information**

Average number of persons who eat at home: 58|__|__|

Number of child aged 10 years old or less: 59|__|__|

Number of liters of oil consumed per month: 60|__|__|.|__|

Number of kg of sugar consumed per month: 61|__|__|.|__|

Are meals taken in front of the TV?

1|__| Never 2|__| Seldom 3|__| Often 4|__| Very often 62|__|

Do you have any diet principles related to religion?

0|__| No 1|__| Yes 2|__| Do not know 63|__|

If yes, does your child abide by the same diet principles than yourself?

0|__| No 1|__| Yes 64|__|

Are there any food allergies in the family?

0|__| No 1|__| Yes 2 |__| Do not know 65|__|

If yes, which one(s) (food excluded by the family)? ……………… 66|__|

................................................................................................................................

................................................................................................................................

**Physical activity of the child**

**Underline in the table below all the activities that the child has done more than 10 times over the previous year, then estimate the time spent by the child for each of the activities underlined** (Do not include school activities)

| Activity | Nb months /year | Nb / week | Nb hours / session |
| --- | --- | --- | --- |
| Basket-ball |  |  |  |
| Bicycle |  |  |  |
| Foot-ball |  |  |  |
| Moringue |  |  |  |
| Danse |  |  |  |
| Gymnastics |  |  |  |
| Hand-ball |  |  |  |
| Walk-hiking |  |  |  |
| Equitation |  |  |  |
| Martial arts |  |  |  |
| Ping pong |  |  |  |
| Skate |  |  |  |
| Roller |  |  |  |
| Climbing |  |  |  |
| Rugby |  |  |  |
| Tennis |  |  |  |
| Swimming in club |  |  |  |
| Swimming for leisure |  |  |  |
| Volley ball |  |  |  |
| Surf |  |  |  |
| Other activity |  |  |  |

How many hours per week your child did practice sports activities:

- last week 67|__|__|.|__| h/week

- last year  68|__|__|.|__| h/week ***(CAUTION : do not include time spent walking and time spent in sport activities taught at school)***

During a typical day, how many hours does your child usually spend:

- sleeping the night 69|__|__|h/day

- naping 70|__|__|h/day - reading, reading, drawing, quiet games (paly cubes, puppet, ...) 71|__|__|h/day

During a typical week, how many hours per day does your child spend watching

TV or playing video games?

**-** a day of the week 72|__|__|h/day

- a day in the week-end 73**|**__|__|h/day

###### During the past 12 months, did your child play in teams of collective sports

###### (football, basketball, …)?

0|__| No 1|__| Yes 2|__| Do not know 74|__|

If yes, number of teams : ……………….…………………………… 75|__|__|

Has your child ever been immobilized in bed or on a chair for more than 2

weeks following an illness or accident?

0|__| No 1|__| Yes 2|__| Do not know 76|__|

If yes, what was his/her age? 77|__|__|month 78|__|__|.|__| year

If yes, how long did this immobilization last? 79|__|__| month 80|__|__| year

In the past year, has your child attended a recreation center on Wednesdays

or during the holidays?

0|__| Never 1|__| Occasionally

2|__| Regularly 3|__| Systematically 81|__|

**School transportation**

How does the child go to school?

1|__| Bus 2|__| Coach 4|__| Walking

8|__| Bicycle 16|__| Other (several choices are possible) 82|__|__|

###### If other (specify).......................................................................................... 83|__|

If by bike, or on foot, how long is the journey: 84|__|__|__| mn

How far is traveled: 85|__|__| km

Health of the child and mother

**Health of the child**

Does your child have any chronic diseases (tooth decay)?

0|__| No 1|__| Yes 2|__| Do not know 86|__|

If yes, which one(s): ................................................................................. 87|__|

.......................................................................................................................

Regular medication?

0|__| No 1|__| Yes 2|__| Do not know 88**|__|**

If yes, drugs: ………………………………….………………………. 89|__|

.................................................................................................................................

###### Are there diabetics persons in your child's immediate family?

0|__| No 1|__| Yes 2|__| Do not know 90**|__|**

If yes:

1|__| Biological father 2|__| Mother

4|__| Brother or sister 8 |__| Other, specifiy: ..........................

....................................................................................................................... 91|__|

HbA1c testing done: 0 |__| No 1 |__| Yes 92**|__|**

Value : 93**|__|__|.|**__|%

**Health of the mother**

Are you diabetic?

0|__| No 1|__| T1D 2|__| T2D 3|__| Do not know 94**|__|**

If T1D or T2D, what is your current treatment:

1|__| diet plan 2|__| tablets 4|__| insulin 95|__|

Age at diabetes onset**:** 96**|__|__|** years

Do you have a familial history of diabetes?

0|__| No 1|__| Yes 2|__| Do not know 97|__|

If yes: 1|__| Father 2|__| Mother

4|__| Brother or sister 8|__| Other (specifiy)  98|__|

.............................................................................................................................. 99|__|

CAUTION: several choices are possible.

Do you have an history of gestational diabetes?

0|__| No 1|__| Yes 2|__| Do not know 100|__|

If yes, for which pregnancy(s)

1|__| Only for the pregnancy of the child surveyed

2|__| Pregnancy(s) previous to that of the child surveyed

4|__| Pregnancy(s) posterior to that of the child surveyed

8|__| All

16|__| Do not know 101|__|__|

If yes, for how many pregancy (G) |__|__| |__| Do not know 102|__|__|

If yes, treatement:

G1 : 0|__|no 1|__|diet only 2|__|insulin 3|__| Do not know 103|__|

G2 : 0|__|no 1|__| diet only 2|__|insulin 3|__| Do not know 104|__|

G3 : 0|__|no 1|__| diet only 2|__|insulin 3|__| Do not know 105|__|

G4 : 0|__|no 1|__| diet only 2|__|insulin 3|__| Do not know 106|__|

G5 : 0|__|no 1|__| diet only 2|__|insulin 3|__| Do not know 107|__|

G6 : 0|__|no 1|__| diet only 2|__|insulin 3|__| Do not know 108|__|

G7 : 0|__|no 1|__| diet only 2|__|insulin 3|__| Do not know 109|__|

G8 : 0|__|no 1|__| diet only 2|__|insulin 3|__| Do not know 110|__|

Do you have other chronic diseases?

0|__| No 1|__| Yes 2|__| Do not know 111|__|

If yes, which ones: ……………………………………………… 112|__|

Regular medication?

0|__| No 1|__| Yes 2|__| Do not know 113|__|

If yes, which ones (other than anti-diabetic, if diabetes): ……………………………………………………………………… 114|__|

HbA1c testing done: 0|__| No 1|__| Yes 115|__|

Value: 116**|__|__|.|**__|%

**sociodemographic characteristics**

**Life and childcare**

Does your child live with both parents?

0|__| No 1|__| Yes 117|__|

If no:

1|__| Alternated childcare 2|__| At his mother’s

3|__| At his dad’s 4|__| At another person 118|__|

Does your child live with his grandparents?

0|__| No 1|__| Yes 119|__|

If yes, are the meals prepared by grandparents?

0|__| No 1|__| Yes 2|__| Do not know 120|__|

Does your child go to school?

0|__| No 1|__| Yes 2|__| Do not know 121|__|

Class:

0|__| GS 2|__| CP 3|__| CE1 4|__|Other 122|__|

If other, specifiy: ……............................................................................... 123|__|

**Characteristics of the mother**

Birth date  124|__|__|/|__|__|/|__|__|__|__|

Number of children   125**|__|__|**

Birth place:

1|__| Réunion 2|__| France mainland 3|__| Other region from OI

4|__|Other 5|__| Do not know 126|__|

Last class attended: ……………………............................................................. 127|__|

What was your age**?** 128**|__|__|**years

Do you have a job: 0|__| No 1|__| Yes 129|__|

If yes, which one: .................................................................................... 130|__|

How many hours per week: 131**|__|__|**h/week

If no, what is your status: ....................................................................... 132|__|

If no, last profession practiced: ………….............................................. 133|__|

What is or was your father’s job:

............................................................................................................................ 134|__|

What is or was your mother’s job:

............................................................................................................................ 135|__|

**Characteristics of the child’s father**

Birth date: 136|__|__|/|__|__|/|__|__|__|__|

Birth place:

1|__| Réunion 2|__| France mainland 3|__| Other region from OI

4|__|Other 5|__| Do not know 137|__|

Last class attended: .............................................................................................. 138|__|

What was your age**?** 139**|__|__|**

Do you have a job: 0|__| No 1|__| Yes 140|__|

If yes, which one: ................................................................................. 141|__|

If no, what is your status: ...................................................................... 142|__|

If no, last profession practiced: ……..................................................... 143|__|

What is or was your father’s job:

............................................................................................................................ 144|__|

What is or was your mother’s job:

............................................................................................................................ 145|__|

**Characteristics of the family**

Currently, is there a smoker at home? 0|__| No 1|__| Yes 146|__|

What is the type of housing? 1|__| Individual 2|__| Collective 147|__|

How much do you estimate your monthly budget for food: 148|__|__|__|__| euros

What is the family monthly income: 149|__|

1|__| less than 400 euros,

2|__| from 400 to less than 800 euros,

3|__| from 800 to less than 1200 euros,

4|__| from 1 200 to less than 2000 euros,

5|__| from 2 000 to less than 4 000 euros,

6|__| from 4 000 to less than 6 000 euros,

7|__| 6 000 euros or more,

8|__| I don’t know,

9|__| I prefer do not answer

**Time at the end of the survey: |__|__|h|__|__|min**

Thank you very much for agreeing to answer this questionnaire.

Do you have any comments: -----------------------------------------------------------------------------------------------------------------------------------------------------------------------------------------------------------------------------------------------------------------------------------------------------------------------------------------------------------------------------------------------------------------------------------------------------------------------------------------------------------------------

Personal observation of the investigator (difficulty to conduct the interview...) :

------------------------------------------------------------------------------------------------------------------------------------------------------------------------------------------------------------------------------------------------------------------------------
