# Supplementary material for: Sexual dimorphism in the association between gestational diabetes mellitus and overweight in offspring at 5-7 years: The OBEGEST cohort study
Source: PLoS One. 2018 Apr 5;13(4):e0195531. doi: 10.1371/journal.pone.0195531 (PMC5886576; doi:10.1371/journal.pone.0195531)
Supplement: S1 Table — This table presents results from a sensitivity analysis performed using child obesity (instead of child overweight including obesity) as dependant variable in the conditional logistic regression models. (DOCX) [file pone.0195531.s007.docx]

**Table S1. Gestational diabetes mellitus exposure and co-factors associated with offspring obesity (BMI ≥ IOTF-30) at 5-7 years by sex**

| **Conditional logistic regression** | **Exposure** | **Boys** (309 pairs) | | |  | **Girls** (291 pairs) | | |
| --- | --- | --- | --- | --- | --- | --- | --- | --- |
|  |  | **OR** | **95% CI** | ***P*** |  | **OR** | **95% CI** | ***P*** |
| **Crude models** |  |  |  |  |  |  |  |  |
| GDM | No | 1.00 | - |  |  | 1.00 | - |  |
|  | Yes | 3.90 | 1.94-7.81 | <0.001 |  | 1.62 | 0.80-3.23 | 0.174 |
| **Adjusted models** |  |  |  |  |  |  |  |  |
| GDM | No | 1.00 | - | 0.027 |  | 1.00 | - | 0.099 |
|  | Yes | 3.18 | 1.14-8.86 |  |  | 0.27 | 0.06-1.28 |  |
| Maternal pre-pregnancy BMI | < 25 kg/m² | 1.00 | - | 0.025 |  | 1.00 | - | 0.012 |
|  | ≥ 25 kg/m² | 4.51 | 1.21-16.83 |  |  | 13.34 | 1.77-100.53 |  |
| Maternal status | Primiparous | 1.00 | - | 0.060 |  | 1.00 | - | 0.738 |
|  | Multiparous | 0.20 | 0.04-1.07 |  |  | 1.37 | 0.22-8.73 |  |
| Maternal age (continuous) | + 5 years | 1.42 | 0.68-2.97 | 0.354 |  | 1.36 | 0.59-3.14 | 0.467 |
| Maternal education | College | 1.00 | - | 0.423 |  | 1.00 | - | 0.350 |
|  | High school | 2.51 | 0.63-10.06 |  |  | 0.38 | 0.05-2.85 |  |
|  | Elementary school | 1.21 | 0.19-7.75 |  |  | 2.33 | 0.29-18.88 |  |

For each sex, two models are presented: one crude (non-adjusted) model and one adjusted model. Figures are odds ratios (OR), 95 % confidence interval of odds ratios, *P*value for global effect. Data are from pairs (1:1) of children (exposed to GDM / unexposed to GDM) matched for sex, gestational age, and birth period. Reference category for dependent variable is BMI < IOTF-30. Missing data distribution (number of missing observations / total number of observations): Crude model for Boys (2/618); Crude model for Girls (0/582); Adjusted model for Boys (11/618); Adjusted model for Girls (13/582). BMI: body mass index. GDM: gestational diabetes mellitus. IOTF: international obesity task force cut-off.
